# The successful accomplishment of nutritional and clinical outcomes via the implementation of a multidisciplinary nutrition support team in the neonatal intensive care unit

**DOI:** 10.1186/s12887-016-0648-0

**Published:** 2016-07-28

**Authors:** Eurim Jeong, Young Hwa Jung, Seung Han Shin, Moon Jin Kim, Hye Jung Bae, Yoon Sook Cho, Kwi Suk Kim, Hyang Sook Kim, Jin Soo Moon, Ee-Kyung Kim, Han-Suk Kim, Jae Sung Ko

**Affiliations:** 1 Department of Pharmacy, Seoul National University Hospital, Seoul, South Korea; 2 Department of Pediatrics, Seoul National University College of Medicine, 101 Daehak-ro, Jongno-gu, Seoul, 110-769 South Korea

**Keywords:** Premature infants, Nutritional support team, Parenteral nutrition, Neonatal intensive care

## Abstract

**Background:**

Nutritional support is critical for preterm infants in the neonatal intensive care unit (NICU). A multidisciplinary nutritional support team (NST) that focuses on providing optimal and individualized nutrition care could be helpful. We conducted a thorough evaluation of clinical and nutritional outcomes in a tertiary NICU following the implementation of an NST.

**Methods:**

This study used a retrospective approach with historical comparisons. Preterm neonates < 30 weeks gestational age or weighing < 1250 g were enrolled. Clinical and nutritional outcomes were compared before and after the establishment of the NST. Medical records were reviewed, and clinical and nutritional outcomes were compared between the two groups.

**Results:**

In total, 107 patients from the pre-NST period and 122 patients from the post-NST period were included. The cumulative energy delivery during the first week of life improved during the post-NST period (350.17 vs. 408.62 kcal/kg, *p* < 0.001). The cumulative protein and lipid deliveries also significantly increased. The time required to reach full enteric feedings decreased during the post-NST period (6.4 ± 5.8 vs. 4.7 ± 5.1 days, *p* = 0.016). Changes of Z-score in weight from admission to discharge exhibited more favorable results in the post-NST period (−1.13 ± 0.99 vs.−0.91 ± 0.74, *p* = 0.055), and the length of ICU stay significantly decreased in the post-NST period (81.7 ± 36.6 vs. 72.2 ± 32.9 days, *p* = 0.040).

**Conclusions:**

NST intervention in the NICU resulted in significant improvements in the provision of nutrition to preterm infants in the first week of life. There were also favorable clinical outcomes, such as increased weight gain and reduced length of ICU stay. Evaluable data remain sparse in the NICU setting with premature neonatal populations; therefore, the successful outcomes identified in this study may provide support for NST practices.

**Electronic supplementary material:**

The online version of this article (doi:10.1186/s12887-016-0648-0) contains supplementary material, which is available to authorized users.

## Background

Multidisciplinary team involvement in providing nutritional support is recommended by the American Society for Parenteral and Enteral Nutrition (ASPEN) and the European Society for Parenteral and Enteral Nutrition (ESPEN) [1, 2]. The team’s main objective is to improve the care of sick patients, and it comprises specialists who focus on the nutritional status and management of patients who require nutritional support [3]. Appropriate nutritional support has been increasingly acknowledged as an integral component of patient management, especially for patients who require intensive care. Pollack et al. [4] reported that critically ill children with associated malnutrition have greater clinical instability and require numerous therapeutic interventions. Previous studies have demonstrated that the involvement of a nutritional support team (NST) in the care of adult patients who required nutritional support was associated with favorable patient safety and cost results [5, 6].

Nutritional support is also essential for preterm infants in the neonatal intensive care unit (NICU). Inadequate nutrient intakes, especially during the first postnatal week, may result in the poor growth of very low birth weight neonates [7]. Despite this knowledge, limited studies have investigated the effects of NSTs on preterm populations in a NICU setting.

Neonatal physiology is very different from that of adult and pediatric populations in that the neonate metabolic pathways are immature and nutrient reservoirs are limited [8]. Moreover, preterm neonates who are managed in a NICU are particularly vulnerable to these problems and often present malnutrition and poor growth [9]. As a result of prematurity, many neonates in the NICU cannot tolerate oral or enteral nutrition (EN) immediately after birth; thus, they rely heavily on parenteral nutrition (PN) for the first few weeks, and the goals of nutritional support are to provide adequate nutrition via PN to avoid nutritional deficits in the immediate post-natal period, initiate enteral feeding as soon as possible and sustain efficient EN intake to promote growth. Thus, the implementation of an NST is especially important in the NICU, where special understanding of the neonate’s physiology, nutritional requirements and tolerance levels are required. To date, published studies have seldom illustrated appropriate and comprehensive outcome measures to evaluate NST practices in preterm infants [10].

In this study, we conducted a thorough evaluation of clinical and nutritional outcomes in an NICU associated with the implementation of a multidisciplinary NST that focused on providing optimal, individualized nutrition care.

## Methods

### Study design and population

This study was approved by the Institutional Review Board of Seoul National University Hospital. This study comprised a retrospective investigation of preterm infants who were admitted to the NICU of Seoul National University Children’s Hospital between January 1, 2009, and August 31, 2010 (a period prior to the establishment of the NST) and between January 1, 2012, and August 31, 2013 (a period subsequent to NST establishment). The inclusion criteria included inborn neonates who were less than 30 weeks gestational age at birth or who had birth weights less than 1250 g. Patients diagnosed with a major congenital anomaly or inborn error of metabolism or who expired within 1 week of life were excluded.

The same feeding protocol for the initiation and advance of enteral feedings was applied throughout the entire study period. As soon as there were no contraindications for feeding, such as hemodynamic instability or abnormal abdomen, the infants began enteral feeding. The feeding volume and the rate of advance of the feedings were practiced according to the internal protocol.

Nutritional and clinical data were collected via reviews of the patients’ electronic medical records. The clinical data for the preterm infants included gestational age, birth weight, mode of delivery, length of ICU stay, and other comorbidities. The infants’ weights at admission and discharge were adjusted for gestational age with reference to the Fenton 2013 preterm growth chart [11]. The nutritional data included the daily intake of energy, protein, lipids and glucose during the first 7 days of life, the number of days to the initiation of enteral feeding, the number of days to reach full enteral feeding (>120 ml/kg/day) and the number of days on PN. The amount of energy intake was calculated by adding both non-protein and protein calories. The parenteral intake and the enteral intake were added to determine the total intake per day. Patients who were received more than 80 kcal/kg/day, which is the minimum number of calories required for growth according to ASPEN, on day 7 were reviewed [12].

### Implementation of the NST

The NST of the Seoul National University Children’s Hospital commenced its work in the NICU in September 2010. Prior to the implementation of the NST, nutrition support was coordinated solely by the attending physician, with intermittent consultation with pharmacists. The aim of the NST was to provide high-quality nutritional support through the enhanced coordination of specialists from various fields, including clinical physicians from the pediatric department, pharmacists, dietitians and nurses. Tasks were based on the ASPEN and ESPEN guidelines and included screening for nutritional risk, identifying patients who require nutritional support, providing adequate nutritional management, educating the hospital staff and auditing practices.

Subsequent to the implementation of the NST, meetings were held once per week to evaluate each patient’s clinical course and nutritional requirements. Changes to be made to the parenteral or EN regimens or additional labs to be analyzed were discussed at this meeting. Future medical plans were shared to ensure that all members were aware of patient management issues. In addition, monthly conferences were held to keep team members abreast of updates and to discuss the current nutritional practices.

Parenteral support was initially managed by the NICU physicians; however, patients who required long-term PN were sometimes referred to the NST pharmacists for customized total parenteral nutrition (TPN). Once a PN referral was made, the pharmacists provided individualized TPN regimens via re-consultations or feedback modulation on a daily basis. Enteral support was managed via protocol; however, EN referrals were made to the NST dietitians.

### Statistical analysis

For comparisons of categorical variables, such as the morbidity rates between the two groups, chi-squared and fisher’s exact tests were performed. Continuous variables were compared via independent *t*-tests. *Z*-scores were evaluated using paired t-tests. Multivariate linear regression analysis was used to investigate the potential confounding factors related to the length of ICU stay. Stepwise selection was used to enter the variables into the regression model according to the default criteria (i.e., inclusion if *p* < 0.05 and removal if *p* > 0.01). A *p*-value < 0.05 was considered statistically significant. All statistical analyses were performed with IBM SPSS Statistics version 22 software (International Business Machines Corp., New York City, NY, USA).

## Results

One hundred seven patients from the pre-NST period and 122 patients from the post-NST period were included in the study. There were no differences in the gestational age at birth (27^+5^ ± 2^+1^ vs. 28^+1^ ± 2^+4^ weeks, respectively) or birth weight (895 ± 260 vs. 952 ± 266 g, respectively) between the pre-NST and post-NST groups (Table 1). Maternal history of chorioamnionitis (25.2 vs. 47.9 %, respectively, *p* < 0.001) and respiratory distress syndrome of the newborn (41.1 vs. 59.0 %, respectively, *p* = 0.007) were more prevalent in the post-NST group than in the pre-NST group. Prenatal steroid use (71.0 vs. 56.6 %, respectively, *p* = 0.023) and treated patent ductus arteriosus (75.7 vs. 60.7 %, respectively, *p* = 0.015) were more prevalent in the pre-NST group than in the post-NST group.

**Table 1:** Demographics of the study population

	Pre-NST (*n* = 107)	Post-NST (*n* = 122)	*p*-value
GA (week)	27^+5^ ± 2^+1^	28^+1^ ± 2^+4^	0.087
Birth weight (g)	895 ± 260	952 ± 266	0.106
Male	51 (47.7)	50 (41.0)	0.310
CS	72 (67.3)	73 (59.8)	0.243
SGA	38 (35.5)	45 (36.9)	0.829
PROM	47 (43.9)	57 (46.7)	0.672
CAM	27 (25.2)	58 (47.9)	< 0.001
Oligohydramnios	21 (19.6)	16 (13.1)	0.182
PIH	15 (14.0)	16 (13.1)	0.842
Maternal DM	4 (3.7)	4 (3.3)	1.000
Prenatal steroids	76 (71.0)	69 (56.6)	0.023
AS 1 min	3.66 ± 2.06	4.07 ± 1.94	0.129
AS 5 min	6.02 ± 1.65	6.25 ± 1.75	0.297
RDS	44 (41.1)	72 (59.0)	0.007
PDA	81 (75.7)	74 (60.7)	0.015

*Abbreviations*: *GA* gestational age, *SGA* small for gestational age, *CS* caesarian section, *PROM* premature rupture of amniotic membrane, *CAM* chorioamnionitis, *PIH* pregnancy-induced hypertension, *DM* diabetes mellitus, *AS* Apgar score, *RDS* respiratory distress syndrome, *PDA* patent ductus arteriosus

Values are presented as means ± SDs or numbers (%)

The daily energy intake during the first week of life improved during the post-NST period (days 2–7), as did the protein intake per day (days 4–6) and the lipid intake per day (days 1–7); there was no difference in the glucose intake per day (independent *t*-test) (Table 2). The cumulative energy delivery during the first week of life increased from 350.2 kcal/kg in the pre-NST period to 408.6 kcal/kg in the post NST period. The proportion of patients who received a minimum of 80 kcal/kg/day on day 7 increased from 42.1 % in the pre-NST period to 63.1 % in the post-NST period (Table 3).

**Table 2:** Nutrition delivered during the first week of life

	PND	Pre-NST (*n* = 107)	Post-NST (*n* = 122)	*p*-value
Energy (kcal/kg)	1	11.99	14.16	0.065
	2	33.27	37.63	0.002
	3	44.12	52.52	< 0.001
	4	54.28	64.47	< 0.001
	5	61.43	74.77	< 0.001
	6	68.9	80.77	< 0.001
	7	76.17	84.31	0.007
	Total	350.17	408.62	< 0.001
Protein (g/kg)	1	0.44	0.56	0.018
	2	1.44	1.52	0.241
	3	1.86	2.01	0.080
	4	2.23	2.48	0.008
	5	2.44	2.77	0.001
	6	2.73	2.98	0.018
	7	2.94	3.03	0.390
	Total	14.08	15.36	0.003
Lipid (g/kg)	1	0	0.13	< 0.001
	2	0.18	0.63	< 0.001
	3	0.6	1.34	< 0.001
	4	1.04	1.88	< 0.001
	5	1.46	2.5	< 0.001
	6	1.87	2.8	< 0.001
	7	2.26	3.12	< 0.001
	Total	7.4	12.42	< 0.001

Values are expressed as means ± SDs. Glucose values (mg/kg/min) were not significantly different

*Abbreviations*: *PND* post-natal day

**Table 3:** Results of nutritional intervention

	Pre-NST (*n* = 107)	Post-NST (*n* = 122)	*p*-value
Energy ≥ 80 kcal/kg on day 7	45 (42.1 %)	77 (63.1 %)	0.001
Lipid initiation (d)	3.4 ± 1.5	1.8 ± 0.8	< 0.001
PN duration (d)	26.5 ± 22.2	22.1 ± 14.3	0.08
Time to initiation of enteral feedings (d)	6.4 ± 5.8	4.7 ± 5.1	0.016
Time to reach full enteral feedings^a^ (d)	23.5 ± 16.2	18.8 ± 12.0	0.015
Weight Z-score at admission	−0.53 ± 1.13	−0.59 ± 1.25	0.690
Weight Z-score at discharge	−1.65 ± 1.01	−1.49 ± 0.99	0.235
Weight Δ Z-score during hospital stay	−1.13 ± 0.99	−0.91 ± 0.74	0.055

Values are expressed as means ± SDs or numbers (%)

^a^Full enteral feeding: ≥ 120 ml/kg/day

All of the patients enrolled in the study received glucose and protein within 24 h of admission according to the basic institutional protocol; however, the time of lipid initiation differed on a case-by-case basis. Subsequent to the implementation of the NST, lipids were initiated earlier (3.4 ± 1.5 vs. 1.8 ± 0.8 days, *p* < 0.001). The initiation of enteral feeding was earlier in the post-NST period compared with the pre-NST period (6.4 ± 5.8 vs. 4.7 ± 5.1 days, *p* = 0.016). The time to reach full enteric feeding was significantly decreased in the post-NST period (23.5 ± 16.2 vs. 18.8 ± 12.0 days, *p* = 0.015), and there was an approximately four-day reduction in the duration of PN, which was borderline significant (26.5 ± 22.2 vs. 22.1 ± 14.3 days, *p* = 0.080). Although there were no differences in the admission *Z*-scores and discharge *Z*-scores between the pre- and post-NST periods, there was a borderline significant decrease in the downward change of *Z*-scores between discharge and admission in the post-NST period (−1.13 ± 0.99 vs.−0.91 ± 0.74, *p* = 0.055 by paired *t*-test).

There were no significant differences in the morbidity rate or the mortality rate between the two groups (chi-square test and independent *t*-test) (Table 4). However, there was a significant reduction in the mean length of ICU stay (81.7 ± 36.6 vs. 72.2 ± 32.9 days, *p* = 0.040). Because of the discrepancies in the basal characteristics between the two groups, multivariate linear regression was used to adjust the effects of potential confounding factors; it revealed that the involvement of the NST independently affected the length of ICU stay (*p* = 0.014). Moreover, NST involvement was the only factor that contributed to the reduction in the length of ICU stay, as the presence of respiratory distress syndrome and patent ductus arteriosus were related to increases in ICU hospitalization (Table 5).

**Table 4:** Clinical outcomes of the study population

	Pre-NST (*n* = 107)	Post-NST (*n* = 122)	*p*-value
BPD	39 (37.5)	49 (41.5)	0.541
IVH (≥ stage 2)	18(16.8)	19 (15.6)	0.469
PVL	11(10.3)	11(9.0)	0.459
NEC	7 (10.7)	11 (9.0)	0.488
ROP (operation)	23 (21.5)	25 (21.4)	0.981
Cholestasis	13 (12.1)	12 (9.8)	0.575
Sepsis	26 (24.3)	36 (29.5)	0.376
Rickets	33 (32.7)	41 (36.9)	0.515
Length of ICU stay (d)	81.72 ± 36.56	72.21 ± 32.89	0.04
Mortality (n)	6 (5.6)	7 (5.8)	0.954

Values are presented as numbers (%) or means ± SDs

*Abbreviations*: *BPD* bronchopulmonary dysplasia, *IVH* intraventricular hemorrhage, *PVL* periventricular leukomalacia, *NEC* necrotizing enterocolitis, *ROP* retinopathy of prematurity

**Table 5:** Multivariate analysis of ICU length of stay

Factors	Parameter	R^2^ value	*p*-value
Intercept	54.831	–	0
PDA	22.738	0.154	< 0.001
RDS	23.973	0.09	< 0.001
Pre/Post-NST group	−10.26659	0.017	0.014

Adjusted for NST group (pre-NST =0, post-NST = 1), PDA, RDS, CAM, Steroid use

*Abbreviations*: *CAM* chorioamnionitis, *RDS* respiratory distress syndrome, *PDA* patent ductus arteriosus

## Discussion

NST practices vary according to the hospital setting, the resources available and the patient characteristics. It is widely accepted that NST involvement is associated with many benefits; however, previous studies on this topic are mainly confined to adult populations. Thus, evaluations of the effects of an NST in a NICU setting must include outcomes appropriate to the neonatal population. However, existing data are too limited to fully describe the advantages of a functioning NST in a NICU [10].

Preterm neonates exhibit physiology and tolerance that differ from adult or pediatric populations. Consequently, they require sufficient energy to sustain life, but this energy must be provided through stepwise advancements to minimize the metabolic disorders that may arise [8, 12]. In general, it is assumed that poor growth in preterm infants primarily reflects inadequate nutrient intake [13]. The ASPEN guideline states that in the presence of adequate protein intake, adequate weight gain occurs at a parenteral energy intake of 80 to 130 kcal/kg/day, which was more achieved by day 7 after NST implementation in the present study [12]. NST intervention reduced the lag time for lipid administration, which enabled more adequate calorie provisions and prevented essential fatty acid deficiencies. Previously, the conventional practice was to withhold lipids in cases of septic conditions, high serum bilirubin levels or pulmonary insufficiency [8]. Studies regarding lipid administration have demonstrated conflicting results [14]; however, current recommendations state that jaundice and sepsis are not absolute contraindications, and most authors now recommend the early initiation of lipids and the advancement to sufficient amounts when tolerated [12, 15–18]. Critical illness induces an increased demand for lipids because of the increased rate of fat oxidation; because children and neonates have limited fat stores, they are susceptible to fatty acid deficiency if they are given a fat-free diet [19, 20].

One of the main functions of a NICU NST that differs from an adult NST is the promotion of appropriate growth [8, 10]. Reassessments of whether patients were maintaining normal growth rates and calorie requirements were routinely performed following the NST implementation. One study evaluated the effect of an NST on the growth rates of a neonatal population [10] and indicated that a greater weight gain from birth to discharge was attained following NST implementation. The advantage of the present study is that weight was modified by age using *Z*-scores, which provide a better assessment of weight change compared to previous studies.

Serious complications resulting from prolonged parenteral access cannot be overlooked. Preterm neonates in a NICU exhibit many risk factors that necessitate long-term parenteral access; thus, the restriction of unnecessary PN use is important. In our study, the mean duration of PN along with the time to reach full enteral feedings was decreased. This difference is likely because of the NST intervention, which included weekly meetings that encouraged active discussions of plans for enteral advancements or various other strategies to improve nutrition access. However, despite the early transition from PN to EN, there were no significant decreases in culture-proven sepsis, rickets or cholestasis.

In the present study, the most notable clinical effect was the reduced mean length of ICU stay by approximately 9 days. Multivariate regression analysis indicated that only the implementation of the NST was significantly and independently associated with a decrease in the length of ICU stay after adjustment for the clinical differences between the two groups. According to previous studies in adult populations, the implementation of early, high-quality nutrition therapy may reduce the length of hospital stay, and several studies also indicate that this approach is cost effective [5, 21].

There are several limitations to the study because it comprises a retrospective analysis with historical comparisons. In the post-NST period, micronutrients, such as trace elements and vitamins, were delivered and monitored more regularly than they were in the pre-NST period; however, comparisons were not possible because of the lack of pre-NST data. Moreover, evaluations of the long-term effects of NST implementation, such as neurodevelopmental outcomes, were not assessed in this study.

## Conclusion

NST intervention in the NICU resulted in significant improvements in the provision of nutrition to preterm neonatal patients. The general nutritional practices improved following NST implementation, and this improvement correlated with important clinical outcomes, such as weight gain or length of ICU hospitalization. Evaluable data remain sparse in the NICU setting with premature neonatal populations; therefore, the successful outcomes identified in this study may provide support for NST practices.

## Abbreviations

ASPEN, American Society for Parenteral and Enteral Nutrition; EN, enteral nutrition; ESPEN, European Society for Parenteral and Enteral Nutrition; NICU, neonatal intensive care unit; NST, nutritional support team; PN, parenteral nutrition; TPN, total parenteral nutrition.

## Additional files

Additional file 1: Raw_data_NST. (XLSX 2379 kb)
